# Generation of Sheep Induced Pluripotent Stem Cells With Defined DOX-Inducible Transcription Factors *via piggyBac* Transposition

**DOI:** 10.3389/fcell.2021.785055

**Published:** 2021-12-16

**Authors:** Moning Liu, Lixia Zhao, Zixin Wang, Hong Su, Tong Wang, Guang Yang, Lu Chen, Baojiang Wu, Gaoping Zhao, Jitong Guo, Zhiqing Yang, Jia Zhang, Chunxia Hao, Teng Ma, Yongli Song, Siqin Bao, Yongchun Zuo, Xihe Li, Guifang Cao

**Affiliations:** ^1^ Inner Mongolia Key Laboratory of Basic Veterinary Medicine, College of Veterinary, Inner Mongolia Agricultural University, Hohhot, China; ^2^ Research Center for Animal Genetic Resources of Mongolia Plateau, College of Life Sciences, Inner Mongolia University, Hohhot, China; ^3^ Inner Mongolia Saikexing Institutes of Breeding and Reproductive Biotechnologies in Domestic Animal, Hohhot, China; ^4^ China State Key Laboratory of Reproductive Regulation and Breeding of Grassland Livestock, College of Life Sciences, Inner Mongolia University, Hohhot, China

**Keywords:** sheep, induced pluripotent stem cells, differentiated potency, transcriptomic, chimera formation

## Abstract

Pluripotent stem cells (PSCs) have the potential to differentiate to all cell types of an adult individual and are useful for studying mammalian development. Establishing induced pluripotent stem cells (iPSCs) capable of expressing pluripotent genes and differentiating to three germ layers will not only help to explain the mechanisms underlying somatic reprogramming but also lay the foundation for the establishment of sheep embryonic stem cells (ESCs) *in vitro*. In this study, sheep somatic cells were reprogrammed *in vitro* into sheep iPSCs with stable morphology, pluripotent marker expression, and differentiation ability, delivered by *piggyBac* transposon system with eight doxycycline (DOX)-inducible exogenous reprogramming factors: bovine OCT4, SOX2, KLF4, cMYC, porcine NANOG, human LIN28, SV40 large T antigen, and human TERT. Sheep iPSCs exhibited a chimeric contribution to the early blastocysts of sheep and mice and E6.5 mouse embryos *in vitro*. A transcriptome analysis revealed the pluripotent characteristics of somatic reprogramming and insights into sheep iPSCs. This study provides an ideal experimental material for further study of the construction of totipotent ESCs in sheep.

## Introduction

Pluripotent stem cells (PSCs), including embryonic stem cells (ESCs) and induced pluripotent stem cells (iPSCs), have unlimited self-renewal capacity and retain the ability to differentiate into nearly all cell types of adult individuals (Navarro et al., 2019). ESCs of mice, rats, humans, porcine, and bovines are established from the inner cell mass (ICM) in the blastocyst (Evans and Kaufman, 1981; Martin, 1981; Thomson et al., 1995; Thomson et al., 1998; Buehr et al., 2008; Gao et al., 2019; Zhao et al., 2021). Since the discovery of reprogramming somatic cells into iPSCs by overexpression of pluripotency-related transcription factors in the mice and humans (Takahashi and Yamanaka, 2006; Takahashi et al., 2007; Yu et al., 2007), reprogramming technology has been wildly used to generate livestock iPSCs, including bovine (Han et al., 2011; Huang et al., 2011; Cao et al., 2012; Lin et al., 2014; Heo et al., 2015; Kawaguchi et al., 2015; Nong et al., 2015; Talluri et al., 2015; Zhao et al., 2017), sheep (Bao et al., 2011; Li et al., 2011; Liu et al., 2012; Sartori et al., 2012; German et al., 2015), goat (Ren et al., 2011; Chu et al., 2015; Sandmaier et al., 2015; Tai et al., 2015; Song et al., 2016; Chen et al., 2017), and porcine (Eccles, 2011; Montserrat et al., 2011; Liu et al., 2012; Wang et al., 2013; Zhang et al., 2015; Mao et al., 2017). Research in iPSCs provides novel insights into the developmental pathway of mammalian development and contributes to regenerative medicine.

Many efforts have been made to generate sheep iPSCs using various culture conditions, different transgenes, and methods. In most reports, adult or fetal fibroblasts were used for reprogramming through lentiviral vectors or retroviral vectors encoding for OCT4, SOX2, KLF4, cMYC (OSKM) (Li et al., 2011; Liu et al., 2012; Sartori et al., 2012; German et al., 2015), OSKM, NANOG, Lin28, SV40 large T antigen, and TERT (Bao et al., 2011). The culture system was based on the characteristic of human and mouse PSCs, and knockout (Dulbecco's Modified Eagle Medium) DMEM or DMEM/F12 was used as basal medium and supplemented with 20% fetal bovine serum (FBS) or KnockOut Serum Replacement (KSR), FGF2, and/or LIF. Most reported iPSCs expressed major pluripotency markers (OCT4, NANOG, and SOX2), retained a normal karyotype, and exhibited differentiation ability to the three germ layers *in vitro* and *in vivo*. Furthermore, Sartori et al. (2012) reported their sheep iPSCs were capable of a low contribution to live-born chimeric lambs, and the expression of exogenous OCT4 could be detected in one lamb skin (*n* = 17) and one dead lamb muscle by RT-PCR (Sartori et al., 2012).

In 2009, the *piggyBac* transposon system was successfully used to induce iPSCs with high transfection efficiency and low risk in clinical application (Woltjen et al., 2009; Yusa et al., 2009). The *piggyBac* transposon system can integrate the carried genome into the host genome and ensure exogenous genes are expressed continuously and stably. Currently, the *piggyBac* transposon system has successfully induced iPSCs in horses (Nagy et al., 2011), porcine (Kim et al., 2019), and bovines (Zhao et al., 2017), but there is no report of using the *piggyBac* transposable system to induce iPSCs in sheep.

Only four Yamanaka factors have been reported not sufficient for the reprogramming of somatic cells from large domestic animals. Besides the four Yamanaka factors, NANOG and LIN28 succeeded in reprogramming human, porcine, and bovine somatic cells (Yu et al., 2007; Gao et al., 2019; Zhao et al., 2021). Also, SV40 large T antigen and human TERT markedly improved human and mouse iPSC reprogramming in efficiency and quality (Mali et al., 2008; Park et al., 2008). The purpose of this study was to establish sheep iPSCs (siPSCs) with greater developmental potential by expressing defined transcription factors (bovine OCT4, SOX2, KLF4, cMYC, porcine NANOG, human LIN28, SV40 large T antigen, and human TERT) through the *piggyBac* transposon system and to provide experimental model for further study on the construction of sheep totipotent ESCs.

## Materials and Methods

### Reprogramming of Sheep iPSCs

Fibroblasts of black-bone sheep (collected 15 days after birth) were seeded in a gelatinized T75 culture flask and cultured in M10 medium. Cultured cells were dissociated using 0.5% trypsin-EDTA (Gibco, 15400-054) and harvested for electroporation at 70% confluence (1.0 × 10^6^ cells per experiment). The M10 medium formulation used was as follows: knockout DMEM (Gibco, 10829-018), 10% FBS (BI, 04-002-1A), 1× MEM non-essential amino acids (Gibco, 10370-021), 1× GlutaMAX (Gibco, 35050-061), and 1× penicillin–streptomycin (Gibco, 11140-050). Transfections were performed using the Amaxa Nucleofector device (Lonza) according to the manufacturer’s protocol (Basic Nucleofector^®^ Kit for Primary Mammalian Fibroblasts, VPI-1001, program U-23), with 6.0 μg DNA: 2.0 μg PB-TRE-bOMSK (bovine OCT4, cMYC, SOX2, and KLF4), 1.0 μg PB-TRE-pNhL (porcine NANOG and human LIN28), 1.0 μg PB-TRE-sLhT (SV40 large T antigen and human TERT), 1.0 μg PB-EF1a-transposase, and 1.0 μg PB-EF1a-rTTA. The PB-TRE-pNhL, PB-EF1a-transposase, and PB-EF1a-rTTA plasmids were provided by Professor Pentao Liu (Gao et al., 2019), and Dr. Lixia Zhao constructed the PB-TRE-bOMSK plasmid (Zhao et al., 2021). The human TERT and SV40 large T antigen cDNA sequences were purchased from Addgene (pBABE-hygro-hTERT, 1773; pSG5 Large T, 9053). The construction of PB-TRE-sLhT was based on PB-TRE-pNhL backbone transposons and was confirmed by sequencing. After transfection, a quarter of the electroconversion product was seeded on STO feeders in M15 medium in 10-cm plates. M15 medium formulation was as follows: knockout DMEM, 15% FBS, 1× penicillin–streptomycin, 1× GlutaMAX, 1× MEM non-essential amino acids, and 2-mercaptoethanol (0.1 mM, Sigma, M6250); LIF (10 ng/ml, Millipore, LIF1001); vitamin C (10 μg/ml, Sigma, 49752); bFGF (10 ng/ml, R&D, 233-FB-025); and doxycycline (DOX) (1.0 μg/ml, Clontech, 631311). The culture medium was changed on alternate days, and colonies were selected on days 7–10 and maintained in M15 medium supplemented with DOX. Colonies with endogenous core pluripotent markers OCT4, SOX2, and NANOG were detected by quantitative reverse transcription PCR (RT-qPCR) assay.

### Culturing Sheep iPSCs

The sheep iPSCs were maintained on STO feeder layers and enzymatically passaged every 2–3 days using TrypLE™ Select (Gibco, 12563-029). The cells were dissociated and centrifuged (300×*g* for 3 min) in K10 medium. K10 medium composition was as follows: DMEM/F12 (Gibco, 11320-033), 10% KSR (Gibco, 10,828-028), 1× penicillin–streptomycin, and 1× MEM non-essential amino acids. After the supernatant was removed, the sheep iPSCs were re-suspended and seeded in M15 medium. All cell cultures used in the study were carried out under conditions of 38.5°C and 5% CO_2_ unless otherwise noted. The sheep iPSCs were frozen once they were ∼80% confluent using cryopreservation medium, which contains 90% FBS and 10% (vol/vol) DMSO (Sigma, D2650).

### Culture Condition Test for Sheep iPSCs

DOX-dependent sheep iPSCs at passage 14 were dissociated in TrypLE™ Select and seeded in a 24-well plate with STO feeder. Cells were cultured in M15 medium supplemented with DOX and then switched to a candidate medium, which removes DOX after 24 h. The candidates included 2i/LIF (Ying et al., 2008), t2iL + Gӧ (Takashima et al., 2014), 4i/L/A (Irie et al., 2015), 5i/L/A (Theunissen et al., 2014), and mouse EPSC medium (mEPSCM) (Yang et al., 2017). Small molecules and cytokines were supplemented as indicated at the following final concentrations: CHIR99021, 0.2 µM (GSK3i, Selleck Chemicals, S2924); PD0325901, 0.2 µM (MEKi, Selleck Chemicals, S1036); Gӧ6983, 5.0 µM (PKCi, Selleck Chemicals, S2911); SP600125, 4.0 µM (JNKi, Selleck Chemicals, S1460); SB203580, 10 µM (p38i, Selleck Chemicals, S1076); Y27632, 10 µM (ROCKi, Selleck Chemicals, S1049); SB590885, 0.25 µM (BRAFi, Selleck Chemicals, S2220); WH4-023, 10 µM (SRCi, Selleck Chemicals, S7565); XAV939, 2.5 µM (WNTi, Selleck Chemicals, S1180); LIF, 10 ng/ml; bFGF, 10–100 ng/ml; and Activin A, 20.0 ng/ml. The medium was refreshed daily. Knockout DMEM, DMEM/F12 (Gibco, 11320-033), and mTeSR™1 (STEM CELL, 85,850) were used as basal medium for culture screening.

### Feeder-Free Culture of Sheep iPSCs

The sheep iPSCs on feeder cells were switched to plates coated with 20 ug/ml fibronectin (Millipore, FC010) and maintained in M15 medium supplemented with DOX. The siPSCs were passaged every 2–3 days and split at a ratio of 1:4. The siPSCs were collected in different passages for the detection of core pluripotent markers by RT-qPCR.

### Embryoid Body Formation Assay of Sheep iPSCs *In Vitro*

Sheep iPSCs were detached from culture plates using TrypLE™ Select, resuspended in M10 medium, and then seeded in a 96-U bottom well plates with ultra-low cell attachment (Corning, 7007). After 3 days in suspension culture, the embryoid bodies (EBs) were transferred and cultured in gelatin-coated plates for an additional 3 days to detect line gene expression by RT-qPCR and an additional 7 days for immunostaining.

### Immunofluorescence for Cells

Sheep iPSCs for immunofluorescence (IF) staining were fixed in 4% paraformaldehyde for 15 min at room temperature after washing with PBS and then permeabilized with IF buffer for 30 min. IF buffer composition was as follows: 0.1% Triton X-100 (Sigma) and 1% BSA in PBS. Cells were incubated with primary antibody at 4°C overnight. After the cells were washed three times with IF buffer for 5 min per wash, secondary antibodies were used to incubate for 1 h at room temperature in the dark and then washed once for 5 min in IF buffer and twice for 5 min in PBS. The cells were then mounted on VECTASHIELD with DAPI (Vector Laboratories). The anti-bodies used are listed in Supplementary Table S1.

### Quantitative Real-Time PCR Analysis

Total RNA was isolated using the RNeasy Mini Kit (Qiagen, 74104). Complementary DNA (cDNA) was prepared using HiScript Q RT SuperMix for qPCR (Vazyme Biotech Co.,ltd, R223-01). RT-qPCR primers are listed in the Key Resource Table. ChamQ Universal SYBR qPCR Master Mix (Vazyme Biotech Co.,ltd, Q711-02) was used for RT-qPCR assays. All RT-qPCR reactions were performed on a Veriti 96-Well Thermal Cycler (Applied Biosystems, United States). To evaluate the expression of the exogenous reprogramming factors, primers were designed to detect the across two adjacent genes in bOSMK, pNhL, and sLhT. The primers used are listed in Supplementary Table S2. Gene expression was determined relative to GAPDH using the ΔΔCt method.

### Generation of Chimera

The H2B-CAG-tdTomato plasmid was transfected into sheep iPSCs using Lipofectamine 2000 (Gibco, 11668027), then screened with hygromycin (100 μg/ml, Gibco, 10687010) after transfection for 48 h. For chimeric contribution to sheep, 10–15 tdTomato^+^ siPSC-4 were injected into sheep blastocysts as described above to detect chimeric contribution *in vitro* and *in vivo*. Half of the injected sheep embryos were cultured *in vitro* in SOF medium and M15 with DOX mixture medium (1:1) at 38.5°C in a 5% CO_2_ atmosphere for 46–48 h for the evaluation of chimerism. The other embryos were transferred to the uteri of pseudopregnant sheep at 7 days post coitus (dpc). After the transplantation for 30 days, pregnancy was diagnosed by ultrasonography. The fetuses were isolated at embryonic stage of days 18–60 to check chimeric contribution.

For chimeric contribution to mice, first, 10–15 tdTomato^+^ siPSC-4 were carefully injected into the early blastocysts of ICR mice using a piezo-assisted micromanipulator attached to an inverted microscope (Zeiss, Eppendorf). One-third of the injected embryos were cultured in KSOM medium (Millipore, MR-020P-5F) and M15 medium combined with the DOX mixture medium (1:1) *in vitro* at 37°C for 16 h to check chimeric contribution *in vitro*. Other embryos were transplanted into the uterus of pseudopregnant ICR female mice at 2.5 dpc. Chimeric embryos were collected at E6.5 to check chimeric contribution *in vivo*. E6.5 mouse embryos (ICR) were dissected from the decidua under stereomicroscopy. Secondly, 10–15 tdTomato^+^ siPSC-4 were injected into the middle post-posterior of E6.5 mouse embryos (ICR) in the same way as above and then cultured *in vitro* in commercial rat serum and M15 with DOX mixture medium (1:1) at 37°C in a 5% CO_2_ atmosphere for 48 h for the evaluation of E8.5 chimerism.

### Cryosectioning and Immunofluorescence Staining

The slides were imaged with a FluoView FV1000 confocal microscope (Olympus). First, E8.5 chimeric embryos were fixed for immunofluorescence assays in 4% PFA for 2 h at room temperature, then dehydrated in 30% sucrose (Sigma) until embryos deposited to the bottom. The E8.5 chimeric embryos were then embedded in a mixture of Thermo O.C.T. compound (SAKURA, 4583) and 30% sucrose overnight at 4°C, then snap frozen in liquid nitrogen. The frozen OCT blocks were sectioned with CryoStar NX50 (Thermo) at 10 µm each section. Sections were first permeabilized with 0.1% Triton and blocked with 5% donkey serum plus 1% BSA followed by incubation with primary antibodies overnight at 4°C. Fluorescence-conjugated secondary antibodies were used to incubate the slides at room temperature for 1 h. Antibodies used are listed in Supplementary Table S1.

### Preparation and Sequencing of the RNA-Seq Library

Total RNA was extracted from cells using TRIzol reagent (Invitrogen, Carlsbad, CA, United States) following the manufacturer’s instructions. After quality control using NanoDrop ND-1000 (NanoDrop, Wilmington, DE, United States), 1–2 µg of RNA was used to extract mRNA according to the NEB Next Poly(A) mRNA Magnetic Isolation Module. The RNA sequencing libraries were then constructed according to the manufacturer’s instructions for the Illumina NEBNext Ultra RNA Library Prep Kit (NEB). The generated libraries were pooled and sequenced on an Illumina NovaSeq™ 6000 following the vendor’s recommended protocol platforms with a 150-bp paired-end mode (sequenced by LC-Bio Technology Co., Ltd. Hangzhou, China).

## Results

### Establishment of siPSC Lines From Sheep Fibroblasts

**FIGURE 1:**
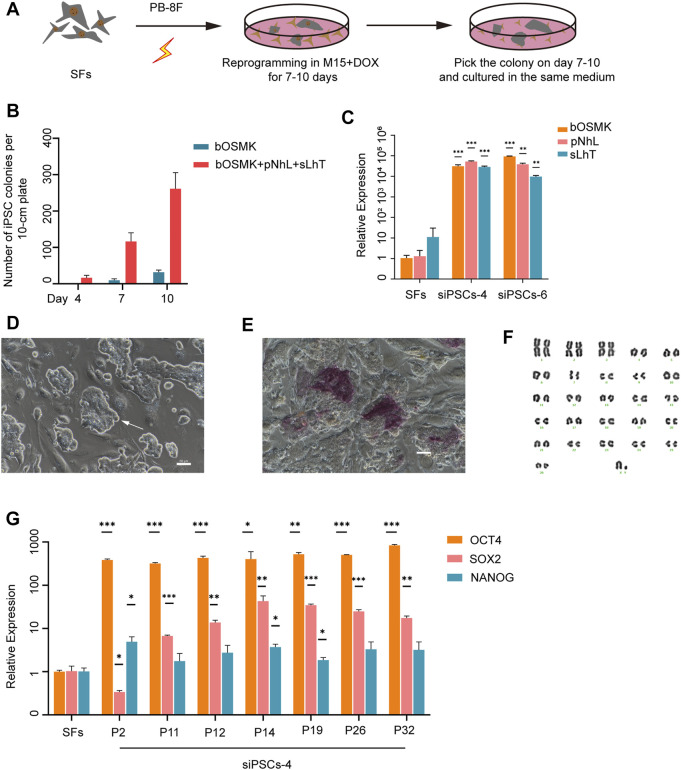
Establishment of sheep induced pluripotent stem cell (iPSC) lines by expressing eight DOX-inducible exogenous reprogramming factors. **(A)** Schematic illustration of reprogramming SFs to iPSCs. PB-8F: bOMSK + pNhL + sLhT. bOMSK (bovine OCT4, cMYC, SOX2, and KLF4 cDNAs), pNhL (porcine NANOG and human LIN28 cDNAs), and sLhT (SV40 large T and human TERT cDNAs). SFs, sheep fibroblasts; DOX, doxycycline. **(B)** The co-expression of LIN28, NANOG, SV40 large T antigen, and human TERT increased the number of reprogrammed colonies from 250,000 SFs along with four Yamanaka factors (*n* = 3 independent experiments). **(C)** The expression level of the eight exogenous reprogramming factors by RT-qPCR in siPSC-4 and siPSC-6. The error bars indicate three independent biological replicates (mean ± SD), *p < 0.05, **p < 0.001, ***p < 0.0001. **(D)** The morphology of siPSC-4 on feeder cells. Scale bar, 50 μm. **(E)** Alkaline phosphatase (AP) staining of siPSC-4 on feeder cells. Scale bar, 50 μm. **(F)** The karyotyping of siPSCs-4 at passage 25 revealed that 31 out of 40 (77.5%) metaphase spreads had a normal karyotype. **(G)** Relative expression of the core endogenous pluripotency genes OCT4, NANOG, and SOX2 in siPSC-4 of various passages. The relative expression was normalized to SFs and the GAPDH housekeeping gene. The error bars indicate three independent biological replicates (mean ± SD), *p < 0.05, **p < 0.001, ***p < 0.0001.

We generated sheep iPSCs by expressing eight DOX-inducible exogenous reprogramming factors, bOMSK (bovine OCT4, cMYC, SOX2, and KLF4), pNhL (porcine NANOG and human LIN28), and sLhT (SV40 large T antigen and human TERT) in fibroblasts of black-bone sheep, delivered *via piggyBac* transposon system (Figure 1A). DOX induction reprogrammed ∼0.1% of the transfected sheep original fibroblasts to primary colonies (Figure 1B). The sheep iPSC colonies were harvested on days 7–10 and passaged in single-cell suspension in serum-containing medium (M15) (Supplementary Figure S1A). The expression of endogenous OCT4 was examined in the second passaged cells by RT-qPCR, and the cells with the highest expression were selected for further study (Supplementary Figure S1B). Then, the cells expressed the eight exogenous reprogramming factors when maintained in M15 in the presence of DOX (Figure 1C and Supplementary Figure S1C). The colonies of cells were dense, with clear boundaries and a high nucleocytoplasmic ratio (Figure 1D). In addition, some cells in the colonies were positive for alkaline phosphatase (AP) (Figure 1E). These cells showed a normal 54 XY karyotype, and the percentage of normal karyotype was 77.5% (31/40) at passage 25 (Figure 1F). The selected cells expressed the endogenous key pluripotent genes OCT4 (POU5F1), SOX2, and NANOG and could be maintained undifferentiated for at least 32 passages when cultured in M15 medium supplemented with DOX (Figure 1G).

### Characterization of siPSCs

**FIGURE 2:**
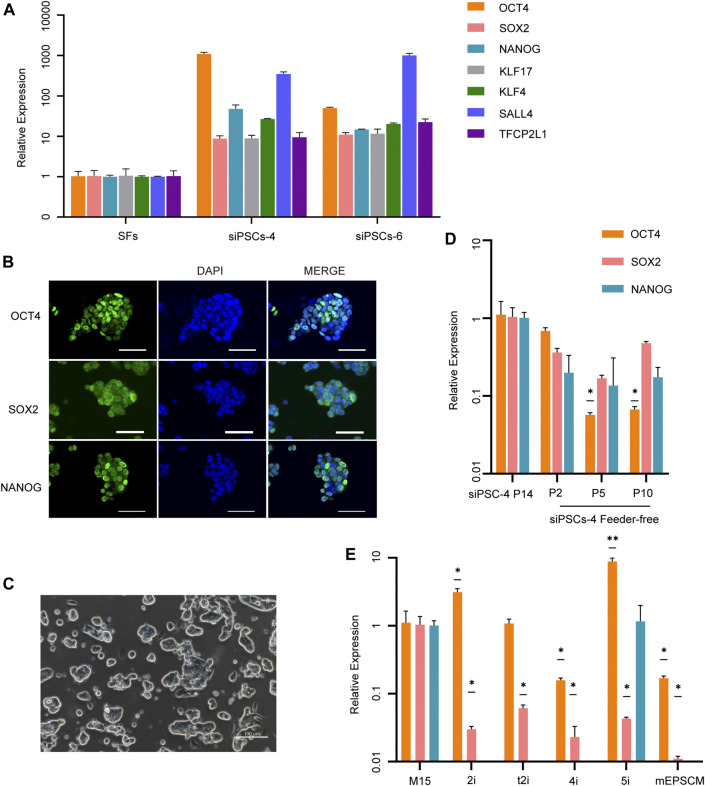
Characterization of siPSCs and their culture conditions. **(A)** The relative expression of key endogenous pluripotency genes in siPSC-4 and siPSC-6. The error bars indicate three independent biological replicates (mean ± SD). **(B)** Immunostaining of OCT4, SOX2, and NANOG in sheep iPSCs. Scale bar, 50 μm. **(C)** The morphology of siPSC-4 without feeder cells. Scale bar, 100 μm. **(D)** Relative expression of OCT4, SOX2, and NANOG in siPSC-4 in feeder-free condition. siPSC-4 for passage 14 were used in the analysis. The error bars indicate three independent biological replicates (mean ± SD), *p < 0.05, **p < 0.001, ***p < 0.0001. **(E)** Quantitative reverse transcription PCR (RT-qPCR) analysis of pluripotency in sheep iPSCs under various culture conditions in the absence of DOX. Cells cultured in M15 media supplemented with DOX for passage 14 were used in the analysis. The error bars indicate three independent biological replicates (mean ± SD), *p < 0.05, **p < 0.001, ***p < 0.0001.

Endogenous pluripotent markers, including OCT4, NANOG, SOX2, KLF17, KLF4, SALL4, and TFCP2L1, were detected in sheep iPSCs by RT-qPCR (Figure 2A), and core pluripotent markers such as OCT4, NANOG, and SOX2 were positive in sheep iPSCs by IF staining (Figure 2B). When cultured without STO feeders, sheep iPSCs still maintained the previous stem cell morphology for at least 10 passages (Figure 2C); however, the cells lost the expression of OCT4 (Figure 2D), suggesting differentiation. We also tested the culture conditions for mouse or human PSCs, including 2i/LIF (Ying et al., 2008), t2iL + Gӧ (Takashima et al., 2014), 4i/L/A (Irie et al., 2015), 5i/L/A (Theunissen et al., 2014), and mEPSCM (Yang et al., 2017). The sheep iPSCs at passage 14 were cultured under these conditions, and the gene expression was examined. We found that upon DOX removal, sheep iPSCs cultured in 2i/LIF, t2iL + Gӧ, 4i/L/A, and mEPSCM were dead after 96 h, and cells cultured in 5i/L/A were dead at 48 h after one passage, concomitant with the loss of pluripotency gene expression (Figure 2E and Supplementary Figure S2A). The results indicated that pluripotency in sheep iPSCs depended on the expression of the DOX-induced exogenous factors in the serum-containing medium and the feeder STO.

### Differentiation Potency of siPSCs *In Vitro*

**FIGURE 3:**
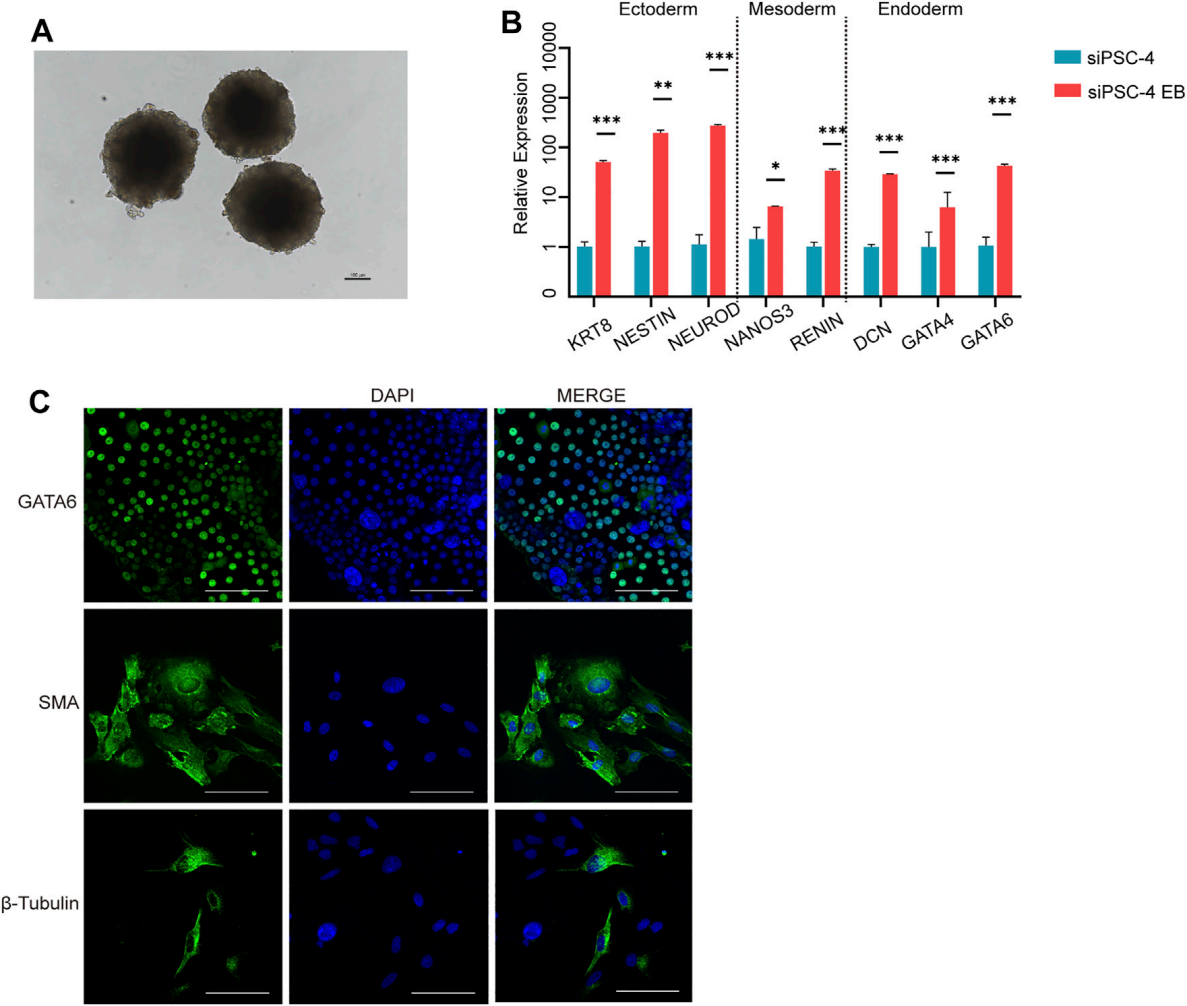
Differentiation of potency of siPSCs *in vitro*. **(A)** The morphology of embryoid bodies (EBs) derived from sheep iPSCs on day 3. Scale bars, 100 µm. **(B)** The relative expression of genes for the three germ layers in cells differentiated from sheep iPSCs by EB. The relative expression was normalized to sheep iPSCs and housekeeping gene GAPDH. The error bars indicate three independent biological replicates (mean ± SD). *p < 0.05, **p < 0.001, ***p < 0.0001. **(C)** Immunostaining of cells differentiated *in vitro* from sheep iPSCs for GATA6 (endoderm), smooth muscle actin (SMA; mesoderm), and β-tubulin (ectoderm). Scale bars, 100 μm.

To test the differentiation capacity of sheep iPSCs *in vitro*, we evaluated EB formation using siPSC-4. Cells formed EBs in 3 days (Figure 3A) and underwent differentiation in M10 medium for 3 days, before analyzing the expression of three germ layer markers. The RT-qPCR results showed that sheep iPSCs were able to differentiate into the three germ layers and expressed the ectoderm (KRT8, NEUROD, and NESTIN), mesoderm (NANOS3 and RENIN), and endoderm markers (GATA4, DCN, and GATA6) (Figure 3B). We then examined the differentiation ability of sheep iPSCs after the EBs were transferred to gelatin-coated plates for continued culture *in vitro*. The attached cells exhibited various types of morphologies and were positive for GATA6 (endoderm), smooth muscle actin (SMA; mesoderm), and β-tubulin (ectoderm) (Figure 3C).

### Transcriptomic Features of siPSCs

**FIGURE 4:**
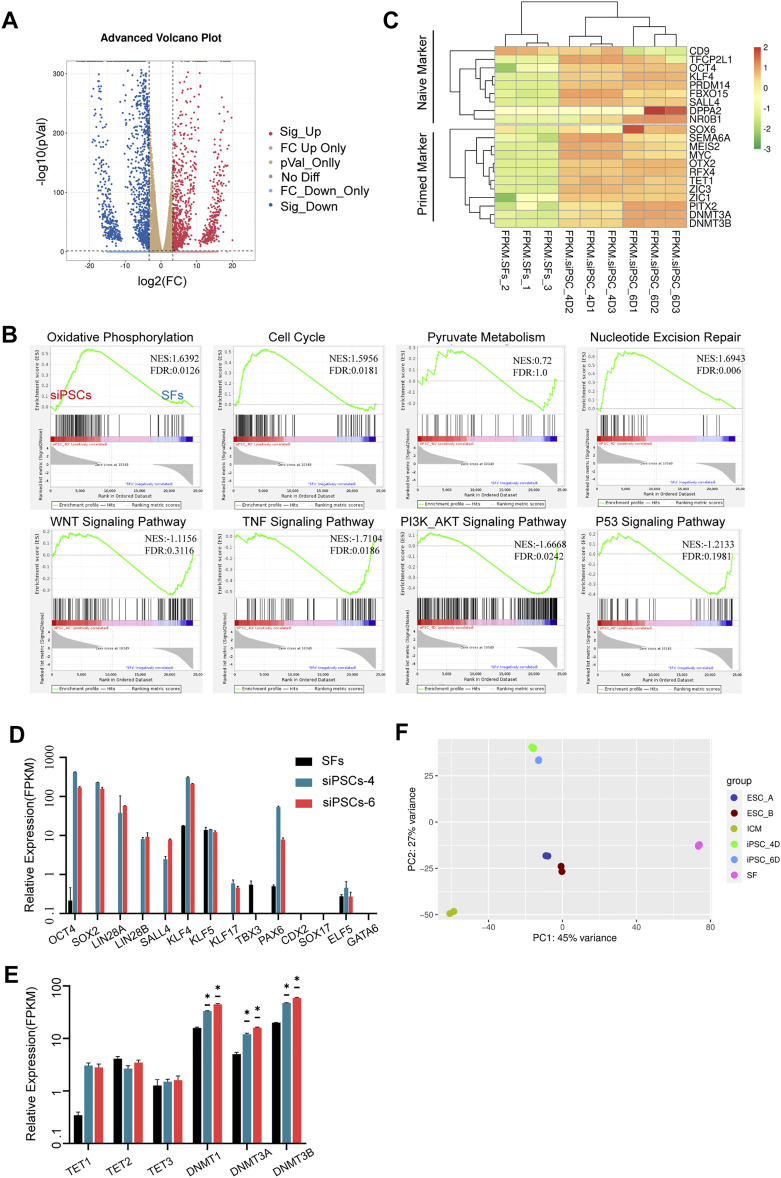
Transcriptomic and epigenetic characteristics of siPSCs. **(A)** Volcano plot of differentially expressed genes for siPSC-4 and SFs. **(B)** Gene set enrichment analysis (GSEA) of siPSC-4 and SFs. The green line shows the enrichment profile. NES, normalized enrichment score; FDR, false discovery rate. **(C)** Heatmap showing a comparison between naïve and primed marker expression in SFs, siPSC-4, and siPSC-6. **(D,E)** The expression of pluripotency genes, lineage genes, and DNA methylation genes in SFs, siPSCs-4, and siPSCs-6 (*n* = 3). *n*, the number of biologically independent samples. *p Val < 0.01. **(F)** Principal component analysis (PCA) of global gene expression (RNA-seq) of sheep iPSCs (iPSC_4D and iPSC_6D) CTFR-sESCs (ESC_A and ESC_B), ICM, and SFs.

To characterize the molecular mechanism of somatic reprogramming and insights into sheep iPSCs, gene expression was determined by transcriptome analyses (RNA-seq). The volcano plot showed that there were 12,740 differentially expressed genes (DEGs) between sheep fibroblasts and sheep iPSCs (*p*-value <0.05, fold change >10), of which 10,270 genes were up-regulated and 2,470 genes were down-regulated (Figure 4A). Compared to sheep fibroblasts, sheep iPSCs presented a significantly higher expression of genes involved in pyruvate metabolism, oxidative phosphorylation, and the cell cycle, whereas genes involved in WNT signaling, TNF signaling, P53 signaling, and PI3K-AKT signaling were significantly overexpressed in the gene set enrichment analysis (GSEA) (Figure 4B). Next, we evaluated the expression of naïve and primed pluripotency markers in sheep iPSCs; some naïve markers, such as KLF4, NR0B1, OCT4, and PRDM14, and primed markers, including DNMT3B, MEIS2, MYC, and ZIC1, were expressed in sheep iPSCs (Figure 4C), suggesting the pluripotency of sheep iPSCs. Corresponding to the results of RT-qPCR, we also found the expression of key pluripotency genes, such as OCT4, SOX2, and SALL4 (Figure 3A; Figure 4D). The DNA methyltransferase genes, DNMT1, DNMT3A, and DNMT3B, were expressed at higher levels in sheep iPSCs (Figure 4E). Sheep iPSCs appeared to have enriched transcriptomic characteristics of CTFR-sESCs, compared to fibroblasts and ICM (Vilarino et al., 2020) in principal component analysis (PCA) (Figure 4F), indicating that sheep iPSCs were reprogrammed in a different state from ICM.

### Development Potential of siPSCs *In Vivo*

**FIGURE 5:**
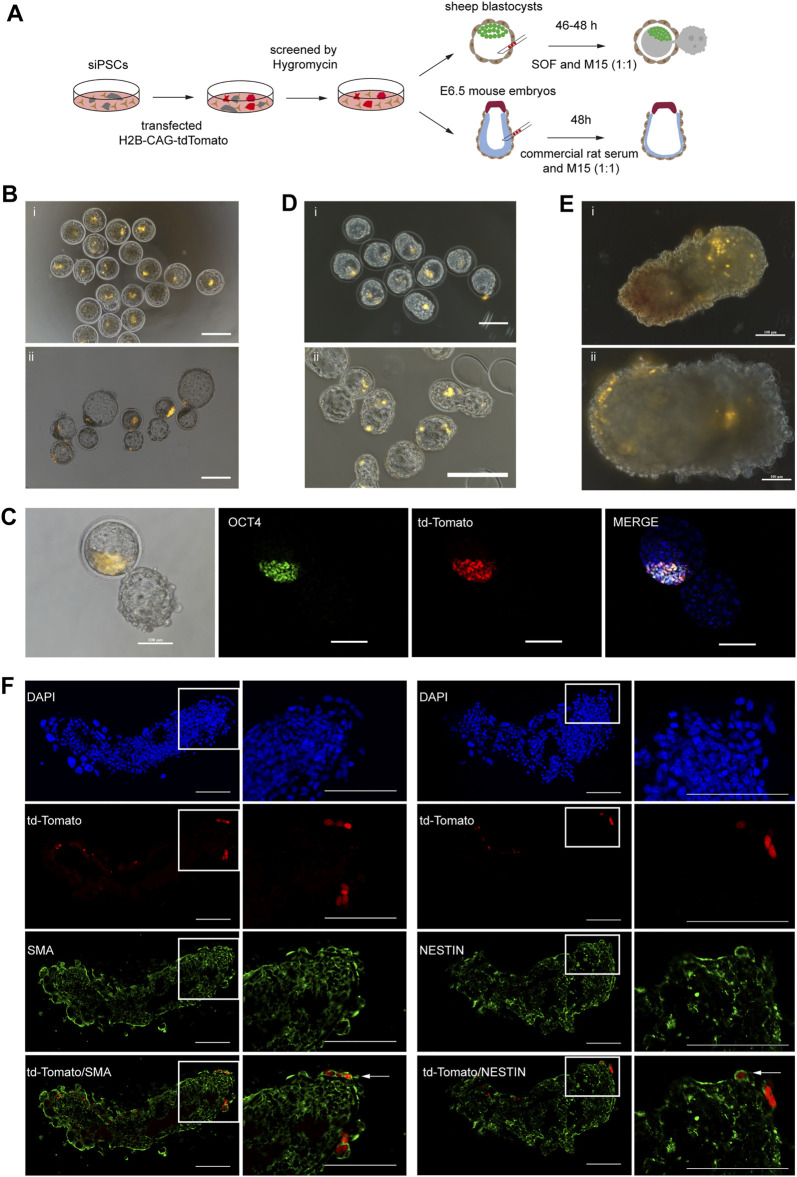
Development potential of siPSCs in chimeras.**(A)** A schematic diagram of chimera experiments using siPSCs.**(B)** The contribution of siPSCs to the development of sheep preimplantation embryos (i, ii). Injection of tdTomato^+^ siPSC-4 (passage 19) into sheep early blastocysts (i). The injected sheep early blastocysts cultured in SOF + M15 (1:1) medium for 46 h (ii). Scale bar, 200 μm. **(C)** Some tdTomato^+^ cells were positive for ICM-specific antibodies (OCT4). Scale bar, 100 μm. **(D)** Contribution of siPSCs in mouse preimplantation embryo development (i, ii). Injection of tdTomato^+^ siPSC-4 (passage 19) in mouse blastocysts (i). Injected mouse blastocysts cultured in KSOM + M15 (1:1) medium for 16 h (ii). Scale bar, 200 μm. **(E)** Analysis of the contribution of siPSCs to E6.5 mouse embryos (i, ii). Injection of tdTomato^+^ siPSCs-4 to E6.5 mouse embryos (i). Injected E6.5 mouse embryos cultured in commercial rat serum + M15 (1:1) medium for 48 h (ii). Scale bar, 100 μm. **(F)** Detection of siPSCs-4 tdTomato^+^ descendants in chimeric embryos at E8.5. tdTomato^+^ siPSC-4 expressed markers of NESTIN (ectoderm) and SMA (mesoderm). Scale bar, 100 μm.

To further investigate the developmental capacity of sheep iPSCs, chimera experiments were performed (Figure 5A). First, the sheep iPSCs were transfected with the H2B-CAG-tdTomato plasmid (Supplementary Figure S2B). We then injected 10–15 tdTomato^+^ siPSCs into the sheep early blastocysts (*n* = 24) and allowed them to develop further for 46 h *in vitro*. tdTomato^+^ cells were detected in ICM at 26% (6 out of 24) (Figure 5B), and some tdTomato^+^ cells were positive for ICM-specific antibodies (OCT4) (Figure 5C). We also injected sheep early blastocysts (*n* = 25) with tdTomato^+^ siPSCs and transferred them to pseudopregnant recipient sheep (*n* = 6). Four recipients became pregnant, and the embryos were harvested on day 11 (*n* = 2) and day 59 (*n* = 1), and we did not detect any siPSCs with tdTomato in the samples (Supplementary Figures S2B, C).

In addition to sheep blastocyst chimera experiments, we also injected tdTomato^+^ siPSCs into mouse early blastocysts to further evaluate the developmental potential of sheep iPSCs. Some of the injected embryos (*n* = 27) were allowed to develop *in vitro*, and others (*n* = 37) were transplanted into 2.5 dpc pseudopregnancy female mice to generate chimeras. tdTomato^+^ cells were detected in both the trophectoderm and the ICM of mouse blastocysts that developed for 16 h *in vitro* (Figure 5D). However, for post-implantation embryos, we did not detect positive tdTomato signals in the E6.5 chimeras (*n* = 27) (Supplementary Figure S2D).

To further assess the incorporation sheep iPSCs in mouse post-implantation epiblasts, we injected 10–15 tdTomato^+^ siPSCs in mid-posterior of E6.5 mouse embryos (Huang et al., 2012; Wu et al., 2015). The embryos were cultured to further assess the incorporation of sheep iPSCs into mouse post-implantation epiblasts *in vitro*, and we identified the descendants of donor siPSCs in chimeric embryos at E8.5 (Figure 5E). We sectioned the cultured embryos and performed IF analysis to detect the expression of the lineage marker of tdTomato^+^ cells. The results revealed that tdTomato^+^ cells could contribute to the ectoderm and mesoderm and expressed markers of NESTIN (ectoderm) and SMA (mesoderm) (Figure 5F).

Collectively, the differentiation data *in vitro* and our siPSCs showed a developmental potential in both sheep and mouse early embryos, but they could not synchronize with the developmental stages of recipient embryo after being transferred *in vivo*.

## Discussion

Although the first sheep iPSCs were reported in 2011 (Bao et al., 2011), cells that do not rely on exogenous genes to maintain pluripotency have not been established. More recently, porcine iPSCs were generated by porcine fetal fibroblasts with eight DOX-inducible transcription factors [porcine (p) OSKM, pNANOG, human (h) LIN28, hRARG, and hLRH1] (Gao et al., 2019). The generated iPSCs were cultured in N2B27 base medium supplemented with vitamin C, activin A, LIF, and small molecule inhibitors of GSK3, non-receptor tyrosine kinase (SRC), and tankyrases/Wnt. This culture condition could be used to “convert” or reprogram porcine iPSCs to EPSCs (Yang et al., 2017; Gao et al., 2019; Yang et al., 2019). Another recent report also verified the strategy developed for culturing of bovine EPSCs (Zhao et al., 2021). However, cells obtained by the porcine or bovine induction system differentiate or die quickly when cultured in M15 with DOX. Still, we found that SV40 large T antigen significantly increased induction efficiency when added to the porcine or bovine induction system [including bovine (b) OSKM or pOSKM, pNANOG, hLIN28, hRARG, and hLRH1]. Furthermore, the addition of hTERT separately to the porcine or bovine induction system could not establish sheep iPSCs. In the meantime, according to the GSEA, sheep iPSCs with SV40 large T antigen induction significantly overrepresented genes in P53 signaling (Figure 4B), and we speculated that SV40 large T antigen plays an essential role in the reprogramming of sheep somatic cells (Ahuja et al., 2005; Bocchetta et al., 2008).

We investigated whether the *piggyBac* transposon system could reprogram sheep somatic cells into iPSCs with eight exogenous reprogramming factors inducible by DOX, bovine OCT4, SOX2, KLF4, cMYC, porcine NANOG, human LIN28, SV40 large T antigen, and human TERT, inspired by the transcription factors used by Bao et al. (2011). The *piggyBac* transposon system worked as well as lentivirus vector, while getting rid of the risk of introducing viruses to host cells (Bao et al., 2011). Furthermore, our first colonies appeared 4 days after transduction, more rapidly than the 14 (Li et al., 2011), 20 (Bao et al., 2011), and 8 days (Sartori et al., 2012) reported previously, and the efficiency of induction was 0.1%, which was more efficient than the previously reported 0.001875% (Liu et al., 2012), suggesting the high efficiency of the *piggyBac* transposon system. In the long-term culture of sheep iPSCs, we found that the status and density of the STO feeder could influence the proliferation rate of sheep iPSCs. When cultured without STO feeder, cells lost OCT4 expression while maintaining cell self-renewal. Culture condition tests indicated that pluripotency in sheep iPSCs depended on the expression of DOX-induced exogenous factors (Yu et al., 2007; Buganim et al., 2014). Altogether, our findings demonstrated that the pluripotency and characteristics of sheep iPSC may depend on the culture conditions used and the cellular environment (Chantzoura et al., 2015), and protocols establishing human and mouse PSCs may not be directly extended to livestock species and need to be optimized further (Roberts et al., 2015; Soto and Ross, 2016).

We further examined the chimeric contribution of sheep iPSCs in the chimera experiment. Sheep iPSCs were able to differentiate and contribute to the ICM of early blastocysts of sheep and mice, but their contribution was limited when transferred *in vivo*. Sartori et al. (2012) pointed out that the low contribution to the chimeras *in vivo* may be due to the culture medium in which sheep iPSCs are grown. In addition, sheep iPSCs showed a chimeric contribution in E6.5 mouse embryos when cultured in commercial rat serum and the M15 medium supplemented with DOX (1:1) *in vitro*. These findings suggested that exogenous pluripotent factors were required to establish and maintain sheep iPSCs in DOX-included conditions, and our reprogramming was incomplete (Buganim et al., 2014; Chantzoura et al., 2015).

Many reports have indicated that human and mouse PSCs can be maintained with IWR-1 supplementation (Sugimoto et al., 2015; Wu and Izpisua Belmonte, 2015). Furthermore, all recent successful attempts in livestock include bovines and porcine, and IWR-1 (or other tankyrase/Wnt inhibitors) was used in isolating and maintaining pluripotent stem cells (Bogliotti et al., 2018; Choi et al., 2019; Gao et al., 2019; Vilarino et al., 2020; Zhao et al., 2021). Furthermore, our data in GSEA showed that sheep iPSCs significantly overrepresented genes in the WNT signaling. It will be interesting to test conditions including IWR-1 in sheep somatic cell reprogramming. According to the PCA of global gene expression (RNA-seq) of siPSCs, CTFR-sESCs, ICM, and SFs, culture conditions with only WNT inhibitors may not be sufficient to maintain pluripotency in the absence of exogenous transgene expression.

In summary, sheep iPSCs established in this study showed stable morphology, pluripotent marker expression, *in vitro* differentiation ability, and interspecies chimeric potential. This study provides an ideal experimental protocol for further study on the construction of sheep totipotent ESCs and may provide a basis for sheep somatic reprogramming studies in the future.

## Data Availability

The datasets presented in this study can be found in online repositories. The names of the repository/repositories and accession number(s) can be found below: All the sequencing data were deposited in the NCBI, Sequence Read Archive (SRA) under the under the accession number PRJNA766237. Data of CTFR-sESCs and ICM are from previously published data (Accession: PRJNA609175) (Vilarino et al., 2020).

## Supplementary Material

Supplementary Figure 1 Reprogramming SFs to DOX-inducible iPSCs. **(A)** The morphology of primary sheep iPSC colonies. Scale bar, 100 μm. **(B)** The expression of endogenous OCT4 in stem cells by RT-qPCR. The error bars indicate three independent biological replicates (mean ± SD); **p* < 0.05, ***p* < 0.001, ****p* < 0.0001. **(C)** The expression of the eight exogenous reprogramming factors by nucleic acid electrophoresis in siPSC-4 and siPSC-6. A, sheep fibroblasts; B, siPSC-4; C, siPSC-6.

Supplementary Figure 2 Culture conditions and development potential in siPSCs chimeras. **(A)** The morphology of sheep iPSCs under several culture conditions in the absence of DOX. These conditions include 2i/LIF, t2iL + Go, 4i/L/A, 5i/L/A, and mEPSCM. Scale bar, 100 μm. **(B)** Relative expression of the core endogenous pluripotency genes OCT4, NANOG, and SOX2 in the labeled siPSCs and unlabeled siPSCs by RT-qPCR. Cells from passage 11 were used to transfect with tdTomato by Lipofectamine 2000, P19 tdTomato+: cells of 8 passages after transfection, and P23 tdTomato+: cells of 12 passages after transfection. The error bars indicate three independent biological replicates (mean ± SD). **(C)** Detection of siPSC-4 tdTomato+ by nucleic acid electrophoresis in embryos from chimaeras. The chimera embryos were harvested on day 59 after embryo transfer. 1: negative control (sheep fibroblasts); 2: heart; 3: liver; 4: lung; 5: kidney; 6: stomach; 7: intestine; 8: limb; 9: skin; 10: positive control (plasmid of tdTomato); 11: H_2_O. **(D)** An analysis of the contribution of siPSCs to chimera embryos (i, ii, and iii). The chimera embryos were harvested on day 11 after embryo transfer. i: embryos developed to day 18; ii: embryos developed to day 17 (conjecture); iii: embryo standstills on day 11 (predicted). Scale bar, 50 μm. **(E)** Detection of siPSC-4 tdTomato+ in post-implantation embryos (E6.5 chimaeras). Scale bar, 100 μm.
