# Supplementary material for: Generation of Sheep Induced Pluripotent Stem Cells With Defined DOX-Inducible Transcription Factors via piggyBac Transposition
Source: Front Cell Dev Biol. 2021 Dec 16;9:785055. doi: 10.3389/fcell.2021.785055 (PMC8716767; doi:10.3389/fcell.2021.785055)
Supplement: Supplementary file 1 [file Table1.DOCX]

**Supplementary Table S1** Antibodies used in this study

| Antigen | Company | Cat No. | Dilution |
| --- | --- | --- | --- |
| Anti-Oct-3/4 | Santa Cruz Biotechnology | Cat# sc-5279 | 1:200 |
| Anti-Sox2 | Millipore | Cat# AB5603 | 1:100 |
| Anti-Nanog | ab80892 | Cat# 14-5761-80 | 1:50 |
| Anti-β-Tubulin | R and D Systems | Cat# MAB1195 | 1:200 |
| Anti-α-SMA | R and D Systems | Cat# MAB1420 | 1:200 |
| Anti-GATA6 | R and D Systems | Cat# AF1700 | 1:200 |
| Anti-NESTIN | Boster Biological Technology | Cat# BA1289 | 1:100 |
