# Supplementary figures and images for: Generation of Sheep Induced Pluripotent Stem Cells With Defined DOX-Inducible Transcription Factors via piggyBac Transposition
Source: Front Cell Dev Biol. 2021 Dec 16;9:785055. doi: 10.3389/fcell.2021.785055 (PMC8716767; doi:10.3389/fcell.2021.785055)

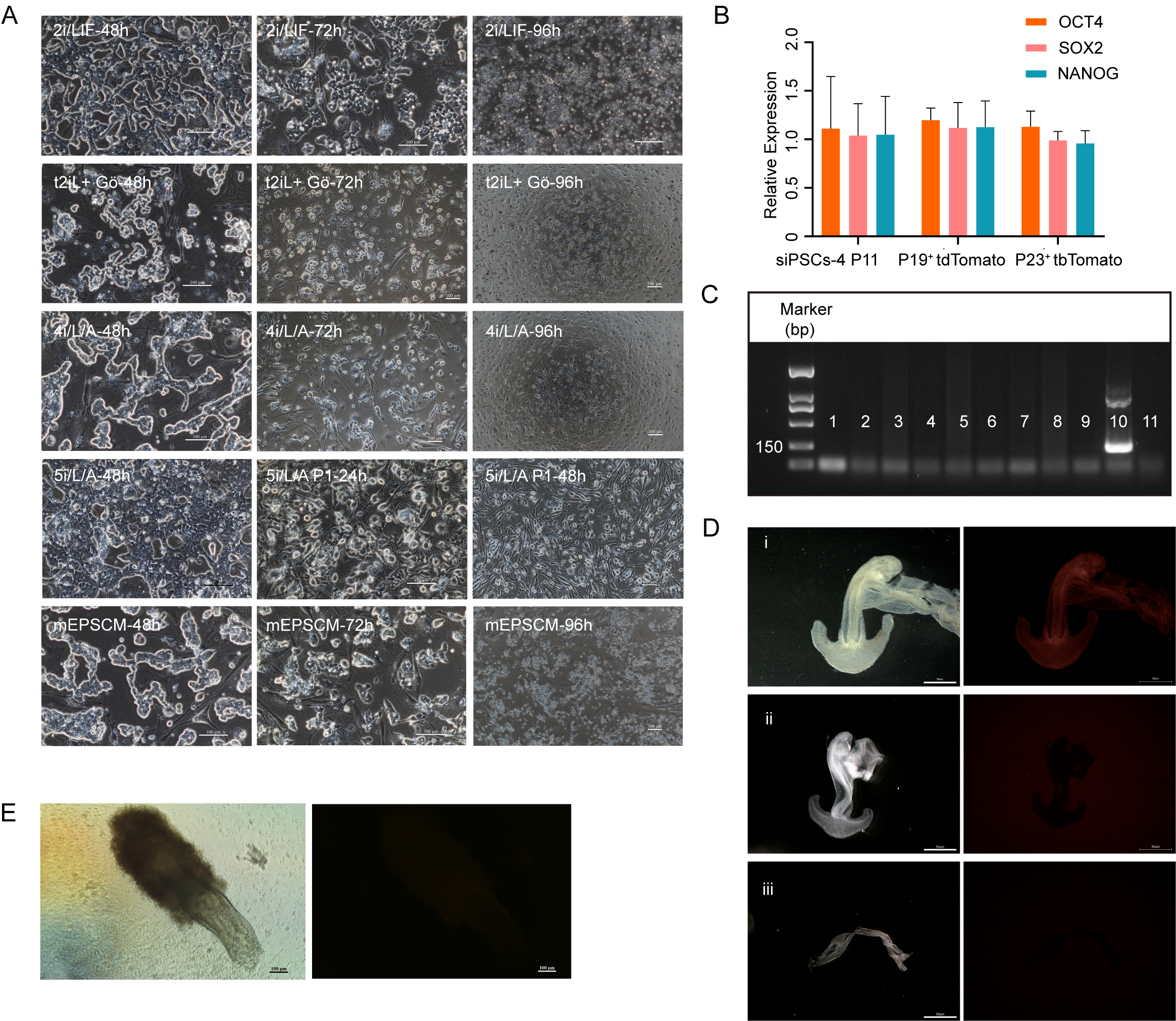

Supplement: Supplementary file 2 [file Image2.TIF]

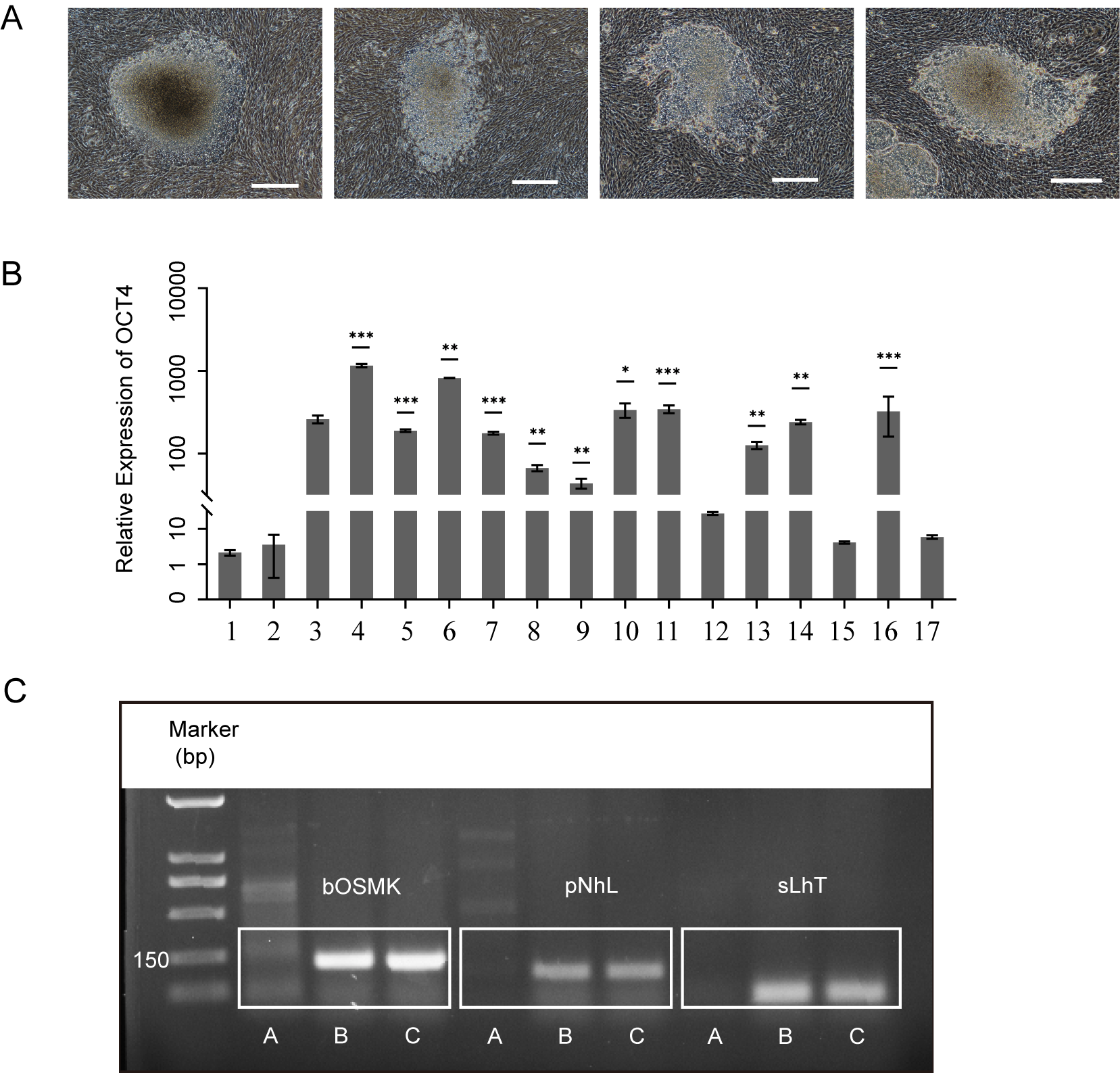

Supplement: Supplementary file 3 [file Image1.TIF]
