# Supplementary material for: Generation of Sheep Induced Pluripotent Stem Cells With Defined DOX-Inducible Transcription Factors via piggyBac Transposition
Source: Front Cell Dev Biol. 2021 Dec 16;9:785055. doi: 10.3389/fcell.2021.785055 (PMC8716767; doi:10.3389/fcell.2021.785055)
Supplement: Supplementary file 4 [file Table2.DOCX]

**Supplementary Table S2** PCR Primer sequences

| Gene name | Forward (5’-3’) | Reverse (5’-3’) |
| --- | --- | --- |
| GAPDH^a^ | acgggaagctcactggcatgg | gccagccccagcatcgaag |
| OCT4^a^ | tacactgtactcttcggtcccatt | agcatcattgaacttcaccttccc |
| NANOG^a^ | caagtatttcagttcccagcagca | tccctcaaactgacacagaaggta |
| SOX2^a^ | tacggtaggagctttgcagaaagt | tgcacgtttgcaactgtcctaaat |
| SALL4 | cacaagtgtcggagcagtgt | gctgctaacagaggcgtcat |
| KLF4 | agggagacggaggagttcaatgatc | aggacgaggaagaggctgatgc |
| KLF17 | cagacagtggatgtgtggct | agactagctggtcagaggca |
| TFCP2L1 | agctgccagatcaaggtgttcaag | tctcataggacggctggtacttctc |
| KRT8 | tacgggacccctggcttcaactac | catcgcgggtctcaatcttcttca |
| NESTIN | cacctcaagatgtccctcagc | tcttcagaaaggttggcacag |
| NEUROD | tctttcaaacacgaaccgtccg | cgtgaaagatggcattgagctg |
| NANOS3 | accttcagtcgcccacctagc | agcattcgccagcaccttgatc |
| RENIN | gtttgatcggcgtaacaatcgc | tggtcctcaaagaagggaagag |
| DCN | aacaatatctctgcaatcggctc | agtttccaagctgaacagcagc |
| GATA4 | ggaagaaagacgacgagcgatgag | cgggtcacgggtagaagagtagag |
| GATA6 | ctcctcttcctcctgctgctctc | atgcgaggcgtagggactgag |
| RT-PCR detection of bOSKM^b^ | ccgcatgttagcagacttcc | gaggattttgaggctgctgg |
| RT-PCR detection of pNL^c^ | tatgtgaaccggtgctagcc | tttggcgagaggggaaagac |
| RT-PCR detection of sLhT^d^ | cccttggacaggctgaactttgag | tcccctccagtgccctttacatc |

Note: a: Primer was quoted from (Bao et al.,2011); b: The primer was designed to detect the whole expression of bovine OCT4 and SOX2 connected by 2A peptide; c: The primer was designed to detect the whole expression of porcine NANOG and LIN28 connected by 2A peptide; d: The primer was designed to detect the whole expression of SV40 Large T antigen and human TERT connected by 2A peptide.

Bao L., He L., Chen J., Wu Z., Liao J., Rao L., et al. (2011). Reprogramming of ovine adult fibroblasts to pluripotency via drug-inducible expression of defined factors. Cell Res. 21(4), 600-608. doi: 10.1038/cr.2011.6
